# Supplementary material for: Profiling of Phenolic Compounds and Triterpene Acids of Twelve Apple (Malus domestica Borkh.) Cultivars
Source: Foods. 2021 Jan 28;10(2):267. doi: 10.3390/foods10020267 (PMC7911497; doi:10.3390/foods10020267)
Supplement: Supplementary file 1 [file foods-10-00267-s001.zip › foods-1069369-supplementary.pdf]

Supplementary materials:

Table S1. Analytical performances of the method used for the quantification of individual phenolic compounds in apple

**Compound Name**	*Linearity*	*R2*	**LOD (mg/100g)**	**LOQ (mg/100g)**	**Precision (%)**	**Recovery (%)**
Caffeic acid	1-50	1.0000	0.11	0.42	3.8	74
Gallic acid	1-50	0.9977	0.41	1.32	4.8	75
Ferulic acid	1-50	0.9980	0.10	0.50	3.3	73
p-Coumaric acid	1-50	0.9980	0. 11	0. 20	2.8	74
p-Hydroxybenzoic acid	1-50	0.9981	0. 10	0. 20	4.1	75
3,4-Dihydroxybenzoic acid	1-50	0.9970	0.10	0.30	3.7	74
t-Cinnamic acid	1-25	0.9980	0. 10	0. 30	4.7	65
Chlorogenic acid	1-50	0.9960	0. 13	0.40	1.0	70
Catechin	1-50	0.9975	0.16	0.50	4.6	75
Epicatechin	1-50	0.9973	0.09	0.22	4.1	67
Quercetin	1-50	0.9960	0.10	0.30	3.9	85
Rutin	1-50	0.9979	0.08	0.25	3.5	76
t-Resveratrol	1-50	0.9950	0.21	0.6	3.8	94

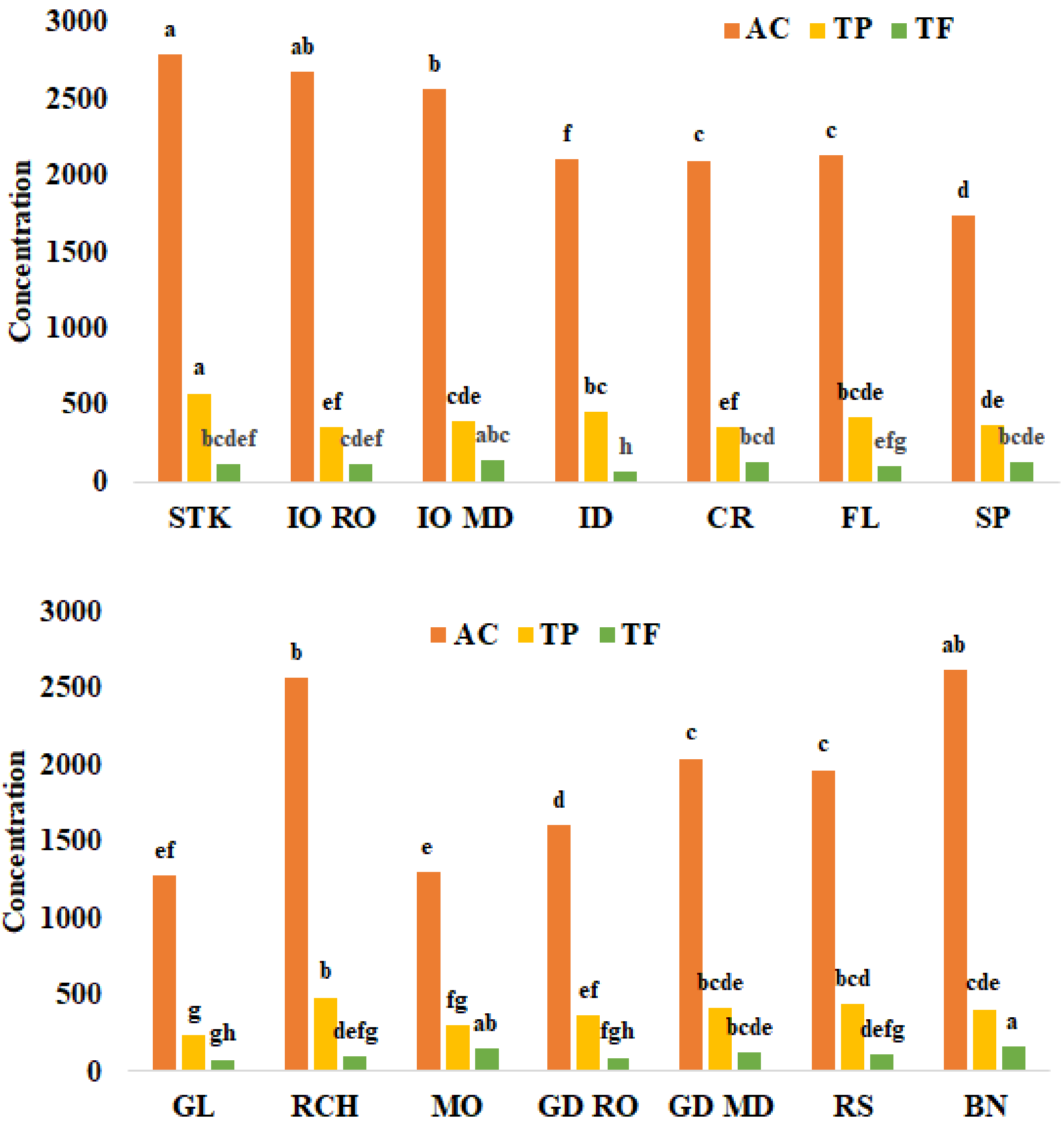

Figure S1. Antioxidant capacity (AC) (μmol Trolox/100g DW)), total polyphenols (TP) (mg GAE/100g DW) and total flavonoids (TF) (RU/100g DW) of different apple cultivars. Different letters denote significant differences according to Duncan test $p \leq 0.05$.
